# The Regeneration of the Virus of Yellow Fever

**Published:** 1887-01

**Authors:** 


### THE REGENERATION OF THE VIRUS OF YELLOW FEVER.

According to promise, we have been favored by Dr. J. McF. Gaston, of this city, with the following interesting and instructive abstract of a notice of the regeneration of the virulence of attenuated culture of the microbe of yellow-fever, by Dr. Dimingos Freire, Professor of Organic Chemistry and Biology in the Medical Faculty of Rio de Janeiro, extracted from the journal *O Paiz*, and translated from the French.

It is very desirable that the attenuated cultures of the xanthogenic microbes shall preserve their activity, and various experiments have been undertaken with this view by the use of heat and electricity. But nature kept the secret of developing its virulence in the hot season and rendering it inert during the cold, which, if discovered, would render it easy to obtain active cultures for the desired researches and demonstrations at all periods of the year.

New experiments have, however, succeeded in perpetuating the virulence of the cultures, and causing them to undergo changes from the energy required for vaccination to the activity which proves fatal.

Inoculation by the hypodermic method, with fifteen grains of the attenuated culture used upon human beings, has been employed with pigeons and chickens, and within three hours afterwards their blood was secured with all due precautions in the sterilized balls.

Subsequently this blood was used to inoculate small birds, and these all died in a period varying from one to seven days.

The autopsy on these birds showed lesions similar to those produced by yellow fever, and presented black matter in the gizzard and in the intestines. The blood of these birds, when examined with the microscope, exhibited the characteristic microbes.

The blood of the chickens and pigeons retained its fatal virulence for sixteen days, after which it became attenuated. In the Cochin China fowls inoculation with the same blood manifested its virulence from two to ten days.

The anatomical lesions confirmed the diagnosis of yellow fever, the liquid in the stomach being very dark and the urine containing albumen.

If the blood of the chicken or pigeon of the first experiment was injected into the bodies of the subjects of the following inoculations, it was observed that the energy of the virus diminished proportionately in the scale of departure.

The attenuated cultures, when inoculated, conferred the immunity of vaccination, and rendered them insusceptible to the action of the fatal virus.

In all these cases, treated with the graduated cultures, the specific microbe, with its ordinary characteristics, was found.

This regeneration of virulence accords with the previous observations of the author and with those of Dr. Range, of France.

### ITEMS.

DR. W. G. ENGLAND, of Cedartown, gave us a pleasant call a few days since.

DR. K. P. MOORE, of Macon, has returned from New York, where he has spent the past several months. Elsewhere we present our readers an interesting letter from him.

WE had a pleasant call a few days since from Dr. T. O. Powell, of Milledgeville, president of the Medical Association of Georgia and superintendent of the State Lunatic Asylum.

MACON MEDICAL SOCIETY.—At the annual election of this society, held Nov. 30th, Dr. E. G. Ferguson was elected president; Dr. H. McHatton, vice-president; Dr. J. Edward Green, secretary and treasurer; Dr. R. O. Cotter, corresponding secretary; Dr. W. C. Gibson, reporter; and Dr. C. H. Hall, librarian.

THE election of Dr. Virgil O. Hardon president of the Atlanta Society of Medicine is an evidence of the esteem in which he is held by the profession of this city. Dr. Hardon has been in Atlanta less than two years; during that time he has made an enviable reputation as a gynæcologist.

CASÇARA CORDIAL.—Dr. R. S. Henry, of Charlestown, W. Virginia, in a paper read before the Medical and Surgical Society of the Kanawha Valley, and published in the *Medical and Surgical Reporter*, Oct. 23, 1886, says of this preparation manufactured by Parke, Davis & Co.: "In addition to its power of disguising the taste of such bitter drugs as quinine, its gentle laxative properties render it peculiarly well adapted for addition as a corrigent to the many preparations which, given alone for any length of time, tend to interfere with the normal action of the bowels, such as the various preparations of iron and opium, than which no others are more frequently indicated and more used by physicians.

THE JOURNAL for February will be an unusually interesting number. Besides the regular stenographic reports of lectures, society and clinical reports, it will contain an able paper from the pen of Dr. Virgil O. Hardon, president of the Atlanta Society of Medicine, on vesical irritation in women. This paper alone is worth the subscription price of THE JOURNAL for one year.

THE CENTURY.—The sales of *The Century Magazine* have gone up over 30,000 copies in six weeks since beginning the Life of Lincoln. A second edition of December was issued on the 15th. A veteran New York publisher predicts that the permanent edition of the magazine will go beyond 300,000 before the completion of the Lincoln history.

A NEW MEDICAL SOCIETY was organized in Birmingham, Ala., in December. It has adopted the name of the Alabama Gynæcological and Surgical Association. Dr. H. N. Rosser, of Birmingham, was elected president; Drs. C. Foxey, of Mobile, and Benj. H. Riggs, of Selma, vice-presidents, and Dr. W. E. B. Davis, of Birmingham, secretary. It promises to be one of the most scientific medical bodies in the South.

THE ATLANTA SOCIETY OF MEDICINE.—At the annual meeting of the Atlanta Society of Medicine, held Tuesday evening, December the 21st, 1886, the following officers were elected for the ensuing year: President, Dr. Virgil O. Hardon; vice-president, Dr. Henry Wile; recording secretary, Dr. F. W. McRae; corresponding secretary, Dr. W. S. Elkin; treasurer, Dr. Hunter P. Cooper; reporters, Dr. James A. Gray, Dr. W. F. Westmoreland, jr., Dr. H. H. Scott.

INJUSTICE TO PHYSICIANS.—A recent number of the Macon *Telegraph* contains the following sensible item to which we direct the attention of our legislators: The judge has his salary, the lawyer his fee, the solicitor his perquisites and the jury its per diem for attending to the business before the courts, but a physician can be hauled out of his practice, whereby he makes his living, placed upon the witness stand and made to give expert testimony free of charge. There is little or no justice in this practice. Clearly the Legislature should arrange for compensation in such cases.

HE WAS FORCED TO TESTIFY.—During a recent murder trial in Macon, Dr. E. G. Ferguson was put on the stand. He refused to testify, saying that his was expert testimony, and physicians in this State received no compensation for such. Judge Simmons said it was true that the State did not make any provision for paying physicians for their expert testimony, but directed the doctor to proceed. The ruling of Judge Simmons is quite different from the following from the New York *Post:* "Judge C. C. Fuller, of Mecosta county, in the case of State of Michigan *vs.* Vanimmans, decided when a physician refused to testify on the ground that the evidence would be expert evidence: 'After many years' study and observation, I decide that a physician's knowledge is his stock in trade, his capital, and we have no more right to take it without extra compensation than we have to take provisions from a grocery without pay to feed the jury. The court rules that the witness is not compelled to testify.' "

THE EFFICACY OF COCA PREPARATIONS.—Dr. H. Lieberman, of Paris, thus writes on this subject:

I desire to state for the benefit of my colleagues the results which I have obtained during my long career as military surgeon by the use of *Vin Coca Mariani.* Briefly stated, I have used it with the greatest success in profound anæmia, resulting from long, arduous campaigns in tropical countries, and in the gastro-intestinal irritation with loss of appetite and dyspepsia, which is such a frequent accompaniment of this condition. Two or three wine-glasses of *Vin Mariani* each day relieved the debility with wonderful rapidity, inasmuch as the tolerance of the stomach for nourishing food and the appetite were restored by its administration. Mariani's wine is vastly superior to the wine of quinia, since the latter by augmenting the gastro-intestinal irritation interferes with the alimentation, and consequently with repair, thereby aggravating the anæmia instead of ameliorating it.

I have also employed it in those cases of chronic alcoholism, fortunately rare in the French army, which follow the abuse of absinthe and strong liquors. Mariani's wine, while producing primarily a certain amount of cerebral stimulation, exercised a predominant sedative effect upon the nervous system. I have, moreover, witnessed the spectacle of hardened drunkards giving up their pernicious habits and returning to a normal condition under the influence of this treatment.

I have also employed Mariani's wine successfully in the treatment of the tobacco habit. A few glasses of the wine, taken in small swallows or mixed with water, were sufficient to replace both pipes and cigars, since the patients obtained the cerebral stimulation which they sought for, albeit unconsciously.

I have also employed it in chronic bronchitis, and even in pulmonary phthisis. Mariani's wine augments the appetite and diminishes the cough in both these conditions. When combating the cough I have given it mixed with water, a wine-glass of the wine to a tumbler of spring water.

Finally, I have employed it in the convalescence following typhoid fever with the greatest success, and this in cases where the irritability of the stomach was so great that no wine, not even Bordeaux, could be tolerated.

To recapitulate, I am convinced that Mariani's wine is the most potent arm which can be placed in the hands of the military surgeon for the purpose of combating the sickness, infirmities and vicious habits engendered by campaigning and the hardships of military life. I will also state that whenever any other than Mariani's preparations of coca were used, the results intended were not produced; quite the contrary; bad effects and even unpleasant complications were noticeable, and to this I call the special attention of the physician.—*N. Y. Medical Monthly.*

